# Artificial intelligence-assisted optimization of *Eichhornia crassipes* extracts and evaluation of their biological activities

**DOI:** 10.1038/s41598-025-16244-6

**Published:** 2025-08-18

**Authors:** Nuh Korkmaz

**Affiliations:** Faculty of Engineering and Natural Sciences, Department of Biology, Osmaniye Korkut Ata University, Osmaniye, Türkiye

**Keywords:** *Eichhornia crassipes*, Extraction optimization, Antioxidant activity, Anticholinesterase effect, Antiproliferative activity, ANN-GA, Phenolic compounds, Biochemistry, Biotechnology, Chemical biology, Chemistry, Drug discovery, Plant sciences

## Abstract

In this research, the extraction conditions for *Eichhornia crassipes* (Mart.) Solms were optimized using Response Surface Methodology (RSM) and Artificial Neural Network–Genetic Algorithm (ANN–GA) techniques to enhance the biological efficacy of the extracts. The optimization focused on three key variables: extraction temperature, duration, and the ethanol-to-water solvent ratio. Through the ANN–GA model, the optimal parameters were identified as 56.85 °C for temperature, 7.62 h for extraction time, and 23.93% for the ethanol/water proportion. The obtained extracts showed statistically significantly higher values ​​compared to RSM in terms of antioxidant capacity (FRAP: 152.89 mg TE/g; DPPH: 121.48 mg TE/g), total phenolic content (TPC: 209.47 mg GAE/g) and flavonoid content (TFC: 263.86 mg QE/g). In addition, ANN–GA extract exhibited high anticholinesterase activity with lower IC₅₀ values ​​against acetylcholinesterase (AChE: 61.69 µg/mL) and butyrylcholinesterase (BChE: 81.40 µg/mL) enzymes. In in vitro tests on A549 cell line, its antiproliferative effect increased significantly in a dose-dependent manner and significant decreases in cell viability were observed especially at high concentrations. LC-MS/MS analyses revealed that pharmacologically important phenolic compounds such as quercetin (10295.26 mg/kg), kaempferol (8656.31 mg/kg) and naringenin (5364.56 mg/kg) were present in high concentrations in the optimized extracts. In conclusion, ANN–GA based extraction approach stands out as an effective method for obtaining phenolic compound rich and biologically effective extracts of *E. crassipes*. These findings indicate that this aquatic plant should be evaluated for its pharmaceutical, neuroprotective and anticancer potential.

## Introduction

Aquatic plants play an indispensable role in freshwater ecosystems, where they contribute significantly to biodiversity, nutrient cycling, oxygen regulation, sediment stabilization, and the maintenance of water quality^1,2^. Their structural and physiological adaptations enable them to thrive in diverse aquatic environments such as lakes, rivers, marshes, wetlands, and artificial reservoirs. Beyond these ecological services, aquatic plants have long been appreciated for their rich secondary metabolite content and associated pharmacological potential. Traditionally used in folk medicine, these species are now under increased scientific investigation for their relevance in drug discovery, natural product chemistry, and environmental health^3,4^.

Among these, *Eichhornia crassipes* (Mart.) Solms—commonly known as water hyacinth—is a free-floating, fast-growing aquatic plant of the Pontederiaceae family, native to tropical South America. It has become naturalized in many parts of the world, especially in nutrient-rich freshwater systems such as ponds, canals, and lakes. Despite its ecological invasiveness, *E. crassipes* is a biologically valuable species due to its high biomass productivity and rich phytochemical profile^5,6^. Numerous studies have reported the presence of phenolic acids, flavonoids, alkaloids, tannins, sterols, and saponins in various plant parts. These compounds have demonstrated a wide range of biological activities, including antioxidant, antimicrobial, anti-inflammatory, anticancer, hepatoprotective, neuroprotective, and larvicidal effects^7–9^. The plant’s allelopathic interactions and ability to suppress the growth of competing organisms further highlight its bioactive potential^10,11^.

In addition to its pharmacological properties, *E. crassipes* holds substantial promise in environmental applications. It has been widely studied for its phytoremediation capabilities, particularly in removing pollutants such as heavy metals (e.g., cadmium, chromium, zinc), phenolic compounds, and cyanide from contaminated water bodies^12,13^. Its high nutrient uptake capacity and rapid growth rate also make it suitable for generating biofertilizers, compost, animal feed, and biogas^14,15^. These multifunctional applications reinforce its value not only as a source of natural therapeutic agents but also as a renewable resource in circular bioeconomy frameworks.

Although the biological effects of *E. crassipes* have been examined using conventional extraction methods, there is limited research applying advanced computational techniques to optimize extraction conditions. Most prior studies lack a systematic approach to improving extraction efficiency and bioactivity outcomes. In this study, we aim to fill this gap by comparing two optimization strategies—Response Surface Methodology (RSM) and Artificial Neural Network–Genetic Algorithm (ANN–GA)—to enhance the extraction of bioactive compounds from *E. crassipes*. The resulting extracts were evaluated for total phenolic and flavonoid content, antioxidant and anticholinesterase activity, and antiproliferative effects on cancer cell lines. Additionally, LC-MS/MS analysis was performed for detailed phytochemical profiling. Unlike previous studies that evaluated *E. crassipes* using conventional extraction approaches without optimization frameworks^12,13^, our study integrates advanced computational methods to systematically enhance extract quality and pharmacological efficacy. To our knowledge, this is the first comparative study using ANN–GA versus RSM to optimize E. crassipes extracts for pharmacological evaluation, offering a novel perspective for future bioresource valorization.

## Materials and methods

### Plant material collection and Preparation

The *E. crassipes* specimens used in this research were collected from Hatay province, Türkiye. The formal identification of *E. crassipes* was undertaken by Dr. Nuh Korkmaz (Osmaniye Korkut Ata University), and a voucher specimen (Voucher number: N.K.−228) has been deposited in the Herbarium of the Department of Biology, Osmaniye Korkut Ata University, Türkiye. All permissions required for the collection of the plants were obtained from the relevant institutions. All experimental research and field studies involving plant collection were performed in accordance with institutional, national, and international guidelines and legislation. Upon arrival at the laboratory, the leaf and flower parts were subjected to drying using a Dalle SS-06 A model dryer set at a constant 50 °C for 6 to 8 h. Once dried, the plant material was uniformly pulverized into a fine powder using a mechanical grinder. These powdered samples were then preserved under suitable conditions for subsequent extraction and biological activity evaluations.

## Extraction procedure method

A full factorial experimental design (FFA) was applied to systematically evaluate the effect of three basic variables in the optimization of the extraction protocol. In this context, three independent variables, namely extraction temperature, process time and solvent composition (ethanol/water ratio), were considered and each was tested at three levels. Experimental studies were carried out using the Soxhlet system and a total of 3³ = 27 different extraction conditions were created. Although Soxhlet extraction is traditionally associated with nonpolar solvents, recent studies have adapted this method for semi-polar systems such as ethanol–water mixtures by implementing temperature-controlled, closed reflux conditions. In the present study, Soxhlet was selected because it allows for continuous solvent cycling and exhaustive extraction, which is suitable for quantitative comparative analysis across multiple variable sets (e.g., temperature, time, and solvent ratio). To address the issue of water evaporation, the system was tightly sealed and fitted with a reflux condenser under constant monitoring, ensuring that the solvent composition remained stable throughout the extraction process. Moreover, all extractions were conducted under controlled temperature conditions (max. 65 °C), which are below the thermal degradation thresholds of most phenolic compounds. Therefore, Soxhlet was preferred for its reproducibility, ease of variable control, and compatibility with the design of factorial experiments in this study. The applied temperature levels were 45 °C, 55 °C and 65 °C; the durations were 5, 10 and 15 h; and the solvent ratios were determined as 0% (pure water), 50% (ethanol/water) and 100% (pure ethanol). For each extraction, approximately 250 mL of solvent was used, and the Soxhlet apparatus enabled 6–8 solvent cycles per hour under consistent reflux. The plant material was ground using a mechanical grinder and passed through a 35-mesh sieve (~ 0.5 mm particle size) to ensure uniform sample texture and improve extraction efficiency.

Nevertheless, we acknowledge that the inclusion of water in Soxhlet extraction introduces a methodological limitation due to its volatility at elevated temperatures. Although reflux control measures were implemented, complete stability of solvent composition cannot be guaranteed. This condition may influence extraction consistency and should be carefully considered in future studies. Using strictly alcoholic or apolar solvents may offer improved reproducibility in Soxhlet-based optimization designs.

The experimental data obtained were analyzed using the Response Surface Methodology (RSM) in order to evaluate multivariate interactions. This process for determining the optimum extraction conditions was supported by a hybrid artificial intelligence model integrated with Artificial Neural Networks (ANN) and Genetic Algorithms (GA) in addition to RSM results. Thus, it was aimed to model the complex relationships between parameters more precisely and to increase process optimization.

## Response surface methodology (RSM)

The aim of this part of the study was to determine the optimal extraction parameters-namely extraction temperature, time, and solvent composition-for maximizing the antioxidant capacity (TAS) of *E. crassipes* extracts. Response Surface Methodology (RSM) was selected as a statistical approach to evaluate the interactions among these variables and to construct a predictive model. This method enables the identification of parameter combinations that yield the highest biological activity, thereby enhancing extraction efficiency in a systematic manner. In this context, extraction temperature, extraction time, and ethanol/water ratio were defined as independent variables, while the TAS value of the obtained extracts was used as the response variable.

The optimization process was carried out using Design Expert 13 (Stat-Ease Inc., Minneapolis, MN, USA) software and was based on the quadratic polynomial modeling approach. Based on the experimental matrix created in Design Expert, a quadratic polynomial model was fitted and validated using R² and ANOVA statistics to identify optimal points for extraction efficiency. This approach is based on a multivariate regression model that takes into account linear, binary interaction and quadratic relationships between variables. This is represented by the following general equation:

$$\:{Y}_{k}={\beta\:}_{k0}+\sum\:_{i=1}^{n}{\beta\:}_{ki}{x}_{i}+\sum\:_{i=1}^{n}{\beta\:}_{kii}{x}_{i}^{2}+\sum\:_{i=1}^{n-1}\sum\:_{j=i+1}^{n}{\beta\:}_{kij}{x}_{i}{x}_{j}$$

In the model, the dependent variable Yk represents the Total Antioxidant Status (TAS) value of the obtained extracts. The terms X_i_ are coded independent variables and represent the extraction temperature, extraction time and solvent ratio (ethanol/water), respectively. The coefficient βk_0_ in the model shows the constant term corresponding to the central point and reflects the initial level of the model. Thanks to this structure, the effects between the variables were systematically analyzed and meaningful parameter combinations were determined for process optimization.

The validity and fit of the model were evaluated based on statistical criteria such as coefficient of determination (R2), analysis of variance (ANOVA) results and p-values. The conditions that maximize the effect of the model on the response variable TAS were calculated from the critical points obtained by taking the derivatives of the created second-degree polynomial equation. In addition, in order to evaluate the effects of the binary interactions between the independent variables on TAS in more detail, three-dimensional response surface plots were used and the obtained findings were interpreted visually. These graphical analyses provided a clearer understanding of the complex relationships between variables and contributed to the determination of optimum extraction parameters. The adequacy of the quadratic model was statistically evaluated using analysis of variance (ANOVA) in Design-Expert software. Model significance, individual term p-values, and the lack-of-fit test were used to assess the validity of the model. Terms with p-values less than 0.05 were considered significant contributors to the response (TAS), while a non-significant lack-of-fit (*p* > 0.05) indicated that the model fit the data well. Coefficient of determination (R²), adjusted R², and predicted R² values were also taken into account to assess model accuracy and predictability.

## Artificial neural network-genetic algorithm (ANN-GA)

In this study, an Artificial Neural Network (ANN) approach was applied to develop a predictive model. In the model, extraction temperature, extraction time and ethanol/water ratio were used as input variables; Total Antioxidant Status (TAS) value of the obtained extracts was used as output variable. The data set used in the modeling process was divided into three subgroups as 80% training, 10% validation and 10% test in order to increase the accuracy and generalizability of the model.

The training of the network model was performed with the Levenberg–Marquardt (LM) optimization method based on the backpropagation algorithm. In order to determine the most suitable network architecture, hidden neurons with the number of neurons in the hidden layer varying between 1 and 20 were tested. In this process, the learning rate and momentum coefficient were kept constant at 0.5; The maximum number of iterations (epochs) was determined as 500, the number of verification checks as 50 and the error tolerance as 1 × 10⁻⁵. Each architectural structure was evaluated with 1000 different trainings and the model with the highest prediction performance was selected as a result of comparative analyses. In the optimal 3-5-1 network structure, the hidden layer employed a tangent sigmoid (tansig) activation function, while the output layer used a linear (purelin) function to ensure continuous output compatibility with the TAS response variable. The Genetic Algorithm was configured with a crossover rate of 0.85 and a mutation rate of 0.02, which were chosen based on preliminary tuning to balance convergence speed and solution diversity. The 3-5-1 architecture was selected because it consistently yielded the lowest error metrics (MSE, MAPE) and the highest R values in comparative trials, demonstrating superior prediction robustness compared to other tested configurations.

The prediction performance of the model was evaluated using two basic statistical measures: mean square error (MSE) and mean absolute percentage error (MAPE). These statistical measures were calculated using the following mathematical formulas:

1 $$\:\text{M}\text{S}\text{E}=\:\frac{1}{\text{n}}\sum\:_{\text{i}=1}^{\text{n}}{\left({\text{e}}_{\text{i}}-{\text{p}}_{\text{i}}\right)}^{2}$$

2 $$\:\text{M}\text{A}\text{P}\text{E}=\frac{1}{\text{n}}\sum\:\left|\frac{{\text{e}}_{\text{i}}-{\text{p}}_{\text{i}}}{{\text{e}}_{\text{i}}}\right|\text{*}100$$

Here, e_i_ represents the experimental values, pi represents the values ​​predicted by the model, and n represents the total number of samples.

The Genetic Algorithm (GA) approach was preferred as the basic calculation method in the optimization process. In this context, different population sizes were tested to evaluate the optimization performance of the algorithm and the effects of parameter settings on the model outputs were systematically analyzed. The probability-based “roulette wheel selection” method was used in the transfer of individuals to the next generations; thus, the probability of selecting solutions with high fitness values ​​was increased. In order to preserve genetic diversity and scan the solution space more effectively, a single-point crossover technique was applied in the crossover phase. In addition, the most appropriate number of iterations for the algorithm to reach the global optimum without showing premature convergence was determined based on the analysis of the created convergence graphs. These graphs provided information about the solution stability and progress trend by monitoring the change in fitness values ​​during each iteration. In order to increase the repeatability and statistical reliability of the obtained results, each GA-based optimization process was performed with 60 independent runs and the obtained data were evaluated collectively. This comprehensive approach increases the overall stability of the algorithm and supports the solution to move towards the global optimum without getting stuck in local minima. While the ANN–GA model demonstrated superior predictive performance and enhanced extract bioactivity compared to the RSM approach, it is important to acknowledge certain limitations. ANN–GA models generally require high computational resources due to iterative training and optimization processes, especially when evaluating numerous configurations. Moreover, there is an inherent risk of overfitting, particularly when the training dataset is limited or the model complexity is high. Although these concerns were mitigated in our study through repeated validations and performance-based architecture selection, they may still affect the model’s generalizability to other datasets or plant systems. Future studies involving cross-validation across independent datasets or external validation using different plant species may help to address these concerns more robustly.

## Extraction for bioactivity

In this study, optimum extraction conditions were determined to maximize biological activity. As a result of Response Surface Methodology (RSM) analyses, optimum extraction parameters were calculated as 53.438 °C extraction temperature, 6.342 h extraction time and 38.827% ethanol/water ratio, respectively. On the other hand, Artificial Neural Network–Genetic Algorithm (ANN–GA) based hybrid optimization approach predicted ideal conditions as 56.845 °C temperature, 7.624 h time and 23.939% ethanol/water ratio. In line with these optimum parameters, extraction processes were carried out using computer-controlled Gerhardt SOX-414 model Soxhlet device. The obtained extracts were analyzed under the specified conditions and their antioxidant, anticholinesterase and antiproliferative biological activities were evaluated comprehensively. Thus, the effect of extraction parameters on biological activity was quantitatively demonstrated and the effectiveness of the optimized method was confirmed.

### Determination of phenolic component profile

The phenolic component profile of the optimized extracts was analyzed using liquid chromatography–tandem mass spectrometry (LC-MS/MS) system. Within the scope of this analysis, both qualitative and quantitative determination of a total of 24 different standard phenolic compounds in the extracts were carried out. The 24 phenolic standards used in this study were selected based on a combination of literature prevalence, relevance to *E. crassipes* phytochemistry, and the availability of high-purity analytical standards. These compounds have previously been reported in aquatic or phenolic-rich plants and are known for their bioactive properties such as antioxidant, anti-inflammatory, neuroprotective, and anticancer effects. Although broader reference libraries can identify a wider range of compounds, this study focused on high-confidence quantification using commercially validated standards with known retention times and fragmentation patterns. This approach was chosen to ensure specificity, reproducibility, and quantitative reliability in comparing extracts optimized by different methods (RSM vs. ANN–GA). Separation of compounds was achieved using a C18 Intersil ODS-4 analytical column with dimensions of 3.0 mm × 100 mm and 2 μm particle size. The column temperature was kept constant at 40 °C throughout the analysis. Chromatographic separation was carried out according to the principle of reverse phase chromatography, and pure water containing 0.1% formic acid was used as mobile phase A and methanol containing 0.1% formic acid was used as mobile phase B. The flow rate was set as 0.3mL/min and 2µL of sample was loaded into the system for each injection. The obtained chromatograms and mass spectra were evaluated for selective and sensitive determination of target phenolic compounds; thus, phenolic contents of optimized extracts were profiled comprehensively. In line with these data, the relationship between the potential biological activities of the extracts and phenolic compound concentrations was interpreted and evaluated.

## Antioxidant activity tests

2,2-diphenyl-1-picrylhydrazyl (DPPH) free radical scavenging test was applied to determine the antioxidant activities of optimized extracts. For this purpose, stock solutions were prepared at a concentration of 1 mg/mL for each extract and dimethylsulfoxide (DMSO) was used as the solvent. 1mL of the prepared stock solutions was taken and mixed with 160µL of DPPH solution prepared at a concentration of 0.267mM. DPPH solution was dissolved in 0.004% methanol solution and a total volume of 4mL was obtained. The prepared reaction mixtures were incubated at room temperature and under light-proof conditions for 30 min. At the end of the incubation period, the absorbance values ​​of the samples were measured spectrophotometrically at 517 nm wavelength. Based on the absorbance data obtained, the DPPH free radical scavenging capacities of the extracts were calculated. Analysis results were expressed in Trolox equivalent (mg TE/g extract) for each extract^16^.

In order to evaluate the antioxidant potential of the optimized extracts, Ferric Reducing Antioxidant Power (FRAP) test was applied. For this purpose, 100µL of stock solution prepared from each extract was mixed with 2mL of FRAP reagent. FRAP reagent was prepared by mixing 300mM acetate buffer (pH3.6), 40mM HCl, 20mM FeCl₃ 6 H₂O and 10mM 2,4,6-tris(2-pyridyl)-S-triazine (TPTZ) solutions at a ratio of 10:1:1. The reaction mixtures were incubated at 37 °C for 4 min and at the end of the incubation period, the absorbance values ​​of the samples were measured spectrophotometrically at a wavelength of 593 nm. The absorbance data obtained were evaluated as an indicator of antioxidant capacity and the results for each extract were calculated in mg Trolox equivalent/g extract (mg TE/g)^16^.

Total antioxidant capacities (Total Antioxidant Status, TAS) of the optimized extracts were determined using a commercial analysis kit developed by Rel Assay Diagnostics (Gaziantep, Türkiye). TAS values ​​obtained as a result of the measurements were expressed in mmol TE/L as Trolox equivalent. Total oxidant level (Total Oxidant Status, TOS) was analyzed using the TOS kit of the same company and the results were reported in µmol H₂O₂ equivalent/L^17,18^.

In addition, the oxidative stress index (OSI) was calculated to evaluate the oxidative stress levels of the extracts. The OSI value was obtained by dividing TOS by TAS and the results were presented in percentage (%). This calculation method allows quantitative comparative evaluation of the oxidative and antioxidative status of the samples and provides important information about the redox balance in biological systems^19^.

## Determination of total phenolic and flavonoid content

1mL stock solution was prepared from plant extracts obtained under optimum extraction conditions and 1mL Folin-Ciocalteu reagent (1:9, v/v) was added to obtain a homogeneous mixture. Then, 0.75mL of 1% sodium carbonate (Na₂CO₃) solution was added to the mixture and the samples were incubated at room temperature for 2 h. At the end of the incubation period, absorbance measurements were performed at 760 nm wavelength and total phenolic substance amounts were calculated in mg/g unit based on the gallic acid standard curve^20^.

Aluminum chloride colorimetric method was used to determine total flavonoid content. In this context, 0.1mL of 10% aluminum nitrate [Al(NO₃)₃], 0.1mL of 1 M ammonium acetate (NH₄CH₃COO), 4.3mL of methanol, 0.5mL of quercetin solution and 0.5mL of extract sample were mixed together. The resulting mixture was left to stand for 40 min and then the absorbance values ​​were measured at 415 nm wavelength. The calculations were based on the quercetin standard curve and the total flavonoid content was expressed in mg/g unit^20^.

### Anticholinesterase activity test

The anticholinesterase activities of the optimized extracts were evaluated based on the colorimetric method developed by Elmann et al.^21^. Galantamine was used as a reference inhibitor in the analyses. Stock solutions were prepared from the extracts at different concentrations in the range of 3.125–200 µg/mL. All biochemical analyses were conducted in a 96-well microplate format. First, 130µL of phosphate buffer solution adjusted to 0.1 M concentration and pH8.0 was added to each analysis well. Then, 10µL of extract solution and 20µL of acetylcholinesterase (AChE) or butyrylcholinesterase (BChE) enzyme solutions were added and the plates were incubated at 25 °C in the dark for 10 min. At the end of the incubation period, 20µL of DTNB [5,5’-dithiobis-(2-nitrobenzoic acid)] solution and 20µL of substrate solution (acetylcholine iodide or butyrylcholine iodide) were added to each well, respectively, to initiate the enzymatic reaction. The absorbances of the reaction products formed as a result of enzyme-substrate interaction were measured spectrophotometrically at 412 nm wavelength. Using the absorbance data obtained, enzyme inhibition percentages were calculated and half-maximal inhibitory concentration (IC₅₀) values ​​(µg/mL) were determined for each extract. Thus, the inhibitory potentials of the optimized extracts on cholinesterase enzymes were quantitatively evaluated.

### Antiproliferative activity test

In this study, the cell growth suppressive (antiproliferative) effects of optimized plant extracts were investigated using human lung adenocarcinoma cell line A549. Within the scope of experimental applications, extracts were prepared and tested at four different concentrations as 25, 50, 100 and 200 µg/mL. When the cells reached 70–80% confluence, they were treated with 3.0mL Trypsin-EDTA solution (Sigma-Aldrich, USA) in order to remove them from the culture surface and transferred back to appropriate culture plates. A 24-hour pre-incubation period was applied to ensure that the cells adhered to the surface and maintained their physiological activity. In the following step, extract solutions at different concentrations were added to the cells and incubated at 37 °C for a 24-hour exposure period. At the end of the period, the culture medium was removed and MTT (3-[4,5-dimethylthiazol-2-yl]−2,5-diphenyltetrazolium bromide) reagent was added to each well at a concentration of 1 mg/mL. The cells were left to incubate for MTT to be reduced by living cells and turn into formazan crystals. Dimethylsulfoxide (DMSO) was added to dissolve the reaction products and the absorbance values ​​of the resulting solutions were measured at a wavelength of 570 nm using a microplate reader system (Epoch, BioTek Instruments, USA)^22^.

### Statistical analysis

The statistical evaluation of all quantitative data obtained in this study was performed using SPSS 21.0 software (IBM Corp., Armonk, NY, USA) integrated into the Windows operating system. In pairwise group comparisons, independent samples t-test was applied in line with the parametric data structure. In data containing three or more groups, one-way analysis of variance (One-Way ANOVA) method was preferred in the analysis of differences between variances. In the event that the ANOVA test yielded significant results, the Duncan method was applied as a multiple comparison test to determine at what levels the differences between groups occurred. In all statistical tests, the significance level was accepted as =0.05, and p-values ​​below this value were considered statistically significant. Although statistical comparisons were conducted using t-tests, ANOVA, and Duncan’s post-hoc analysis with p-values indicating significance, effect size measures such as Cohen’s d and confidence intervals (CIs) for key parameters (e.g., IC₅₀, TPC, TFC) were not calculated in the current study. These statistical metrics can provide deeper insights into the magnitude and precision of observed differences. Their inclusion is strongly recommended for future studies to improve the interpretability and reproducibility of quantitative comparisons.

### Results and discussions

#### Optimization of extraction conditions

In this study, three main extraction parameters affecting the total antioxidant capacity (TAC) of extracts were investigated comprehensively. The variables investigated were determined as extraction temperature (45, 55 and 65 °C), extraction time (5, 10 and 15 h) and solvent system (ethanol/water ratio: 0%, 50% and 100%). The TAS values ​​of the samples obtained as a result of extractions performed with different combinations of these three parameters are reported in detail in Table 1. The obtained data provide the opportunity to comparatively evaluate the effects of extraction conditions on total antioxidant activity and form the experimental basis for process optimization. Thus, an important preliminary analysis was carried out to identify the conditions providing the highest biological activity.

**Table 1:** TAS values of the extracts obtained in the study. ^*^Means in the same column with different superscript letters are significantly different (*p* < 0.05) according to duncan’s multiple range test.

Experiment number	Extraction temperature (°C)	Extraction time (h)	Ethanol/water ratio (%)	TAS (mmol/L)
**1**	45	5	0	6.703 ± 0.017^fg^
**2**	45	10	0	7.088 ± 0.055^i^
**3**	45	15	0	6.006 ± 0.040^cd^
**4**	45	5	50	6.721 ± 0.027^g^
**5**	45	10	50	7.124 ± 0.031^i^
**6**	45	15	50	5.959 ± 0.043^c^
**7**	45	5	100	6.652 ± 0.033^f^
**8**	45	10	100	7.110 ± 0.064^i^
**9**	45	15	100	6.017 ± 0.043^d^
**10**	55	5	0	7.748 ± 0.034^j^
**11**	55	10	0	8.570 ± 0.024^k^
**12**	55	15	0	6.856 ± 0.021^h^
**13**	55	5	50	7.757 ± 0.043^j^
**14**	55	10	50	8.539 ± 0.029^k^
**15**	55	15	50	6.852 ± 0.039^h^
**16**	55	5	100	7.765 ± 0.028^j^
**17**	55	10	100	8.561 ± 0.029^k^
**18**	55	15	100	6.847 ± 0.012^h^
**19**	65	5	0	5.759 ± 0.030^b^
**20**	65	10	0	6.153 ± 0.029^e^
**21**	65	15	0	4.756 ± 0.028^a^
**22**	65	5	50	5.744 ± 0.018^b^
**23**	65	10	50	6.164 ± 0.024^e^
**24**	65	15	50	4.745 ± 0.033^a^
**25**	65	5	100	5.734 ± 0.022^b^
**26**	65	10	100	6.158 ± 0.021^e^
**27**	65	15	100	4.750 ± 0.026^a^

A one-way analysis of variance (ANOVA) was conducted to examine differences in TAS values among 27 extraction condition combinations (3 temperatures × 3 extraction times × 3 ethanol/water ratios). The analysis indicated a statistically significant difference in TAS across groups, F(26, 54) = 3205.78, *p* < 0.001, η² = 0.91. The analysis results showed that total antioxidant capacity (TAS) reached the highest level at 55 °C extraction temperature and 10 h processing time. At this optimal temperature and time combination, TAS levels measured depending on the solvent system were recorded as 8.570 ± 0.024, 8.539 ± 0.029 and 8.561 ± 0.029mmol/L at 0%, 50% and 100% ethanol/water ratios, respectively. These results show that the relevant parameter combination provides the most suitable conditions in terms of extraction efficiency. On the other hand, in extractions carried out at lower temperatures such as 45 °C, TAS values ​​were found to vary between 5.959 ± 0.043mmol/L (15 h, 50% ethanol) and 7.124 ± 0.031mmol/L (10 h, 50% ethanol). These data indicate lower antioxidant activity and relatively stable redox capacity. On the other hand, a significant decrease was observed in the extraction processes carried out at 65 °C; TAS levels remained between 4.745 ± 0.033mmol/L (15 h, 50% ethanol) and 6.164 ± 0.024mmol/L (10 h, 50% ethanol). This decrease indicates that antioxidant compounds may undergo thermal degradation under high temperature conditions. In addition, increasing the extraction time to 15 h negatively affected the TAS values ​​in all temperature groups. This decreasing trend was observed more sharply, especially at 65 °C. On the other hand, while the differences in the solvent ratio at 55 °C conditions did not create a statistically significant change on the TAS values, increasing the ethanol ratio at 65 °C showed a slight balancing effect in limiting the TAS decrease. In this study, two different optimization strategies were applied based on the experimental data set obtained. In the modeling process carried out within the framework of Response Surface Methodology (RSM), linear, two-factor interactive (2FI), quadratic and cubic regression models were compared statistically. As a result of the evaluations, the quadratic model determined at the level of R2 = 0.980 was selected as the most appropriate model due to having the highest fit coefficient. This finding shows that 98% of the variation in the dependent variable total antioxidant capacity (TAS) value is explained by independent variables such as extraction temperature, extraction time and solvent ratio. The high explanatory level of the model reveals that the trends in the obtained experimental data are reliably represented and the relationships between the variables can be modeled correctly. Therefore, it was confirmed that the selected quadratic model has statistical validity and high predictive power in the optimization process. The mathematical expression of the quadratic polynomial model obtained as a result of the multivariate regression analysis performed to predict the total antioxidant capacity (TAS) levels of the *E. crassipes* species is given below. This model numerically represents the effects of extraction process parameters on TAS and provides a powerful tool to predict the behavior of the system.

$$\begin{array}{l} \:TAS = 8.37 - 0.523\:{X_1} - 0.002\:{X_2} - 0.432\:{X_3} - 0.0004\:{X_1}{X_2}\\ - 0.074\:{X_1}{X_3} - 0.004\:{X_2}{X_3} - 1.65\:X_1^2 - 0.001 - 0.973\:X_3^2 \end{array}$$

**Fig. 1:**
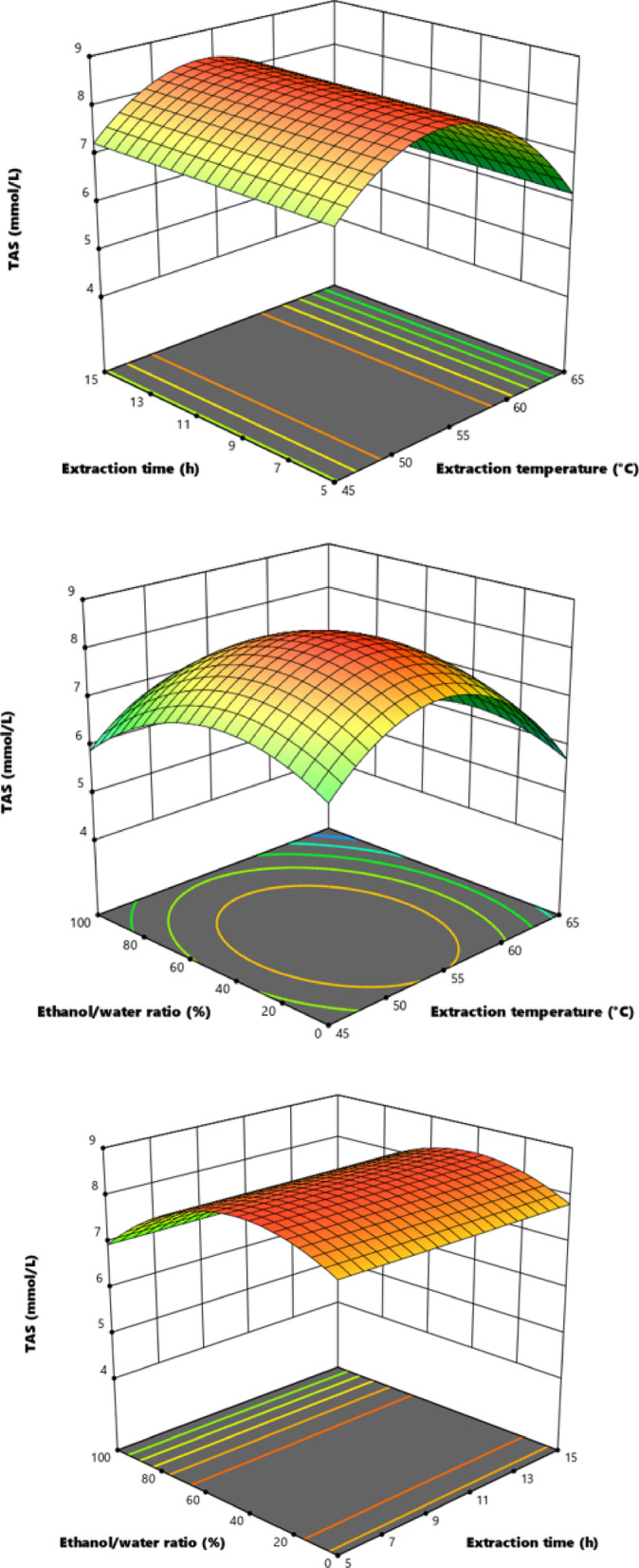
Response surface plots.

In this equation, X₁, X₂ and X₃ represent the extraction temperature, extraction time and ethanol/water ratio, respectively. Response surface graphics visually reflecting the total antioxidant activity (TAS) values ​​of the *E. crassipes* species are given in Fig. 1.

**Fig. 2:**
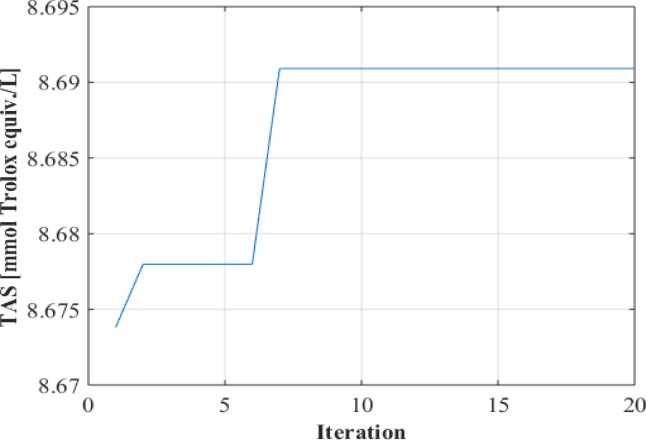
Convergence graph.

Within the scope of the AI-supported optimization process, the Artificial Neural Network (ANN) algorithm was used in modeling the experimental data set. As a result of the comparative evaluation of different network architectures, the structure with the highest prediction performance was determined and integrated with the Genetic Algorithm (GA) in order to achieve advanced optimization. In this context, the ANN model with a 3-5-1 architecture and five neurons in a single hidden layer stood out with its superior prediction ability. The accuracy level of the model was quantitatively evaluated with various performance metrics. The mean squared error (MSE) value was calculated as 0.003, the mean absolute percentage error (MAPE) as 0.160% and the correlation coefficient (R) as 0.999. These results show that the model has both high prediction ability and strong generalizability; in this context, it statistically confirms the suitability of the selected structure for the modeling process. In the next stage of optimization, Genetic Algorithm was applied based on the ANN model that showed the best performance. Different population sizes were tested to determine the effectiveness of GA parameters; as a result of these analyses, the population size at which the algorithm worked most efficiently was determined as 10. The success of the GA optimization process is visually supported by the convergence graph created and presented in Fig. 2. The relevant graph clearly shows that the algorithm reached the global optimum steadily after approximately 7 iterations.

### Antioxidant activity

Aquatic plants are also important from a pharmacological perspective due to their high levels of biologically active compounds as well as their ecosystem services^23^. Phenolic compounds and flavonoids found in plants provide a protective effect against free radicals by exhibiting strong antioxidant properties^24^. Therefore, aquatic plants have the potential to be evaluated as natural antioxidant sources. In our study conducted within this scope, the antioxidant potentials of optimized extracts obtained from *E. crassipes* were determined and the findings obtained are presented in detail in Table 2.

**Table 2:** Antioxidant parameters of ***E. crassipes***. ^*^Values in the same row with different superscript letters are significantly different (*p* < 0.05) according to independent t-test. The fitness function used was the total antioxidant status (TAS) prediction error minimization based on MSE. All cohen`s D were observed very high (> 1.4).

Parameters	RSM extract Mean ± SE [95%CI]	ANN-GA extract Mean ± SE [95%CI]
**TPC (mg GA Equi/g)**	148.880 ± 1.571 [147.102, 150.658]^a^	209.467 ± 3.590 [205.405, 213.529]^b^
**TFC (mg QE Equi/g)**	198.667 ± 2.028 [196.372, 200.962]^a^	263.867 ± 2.643 [260.876, 266.858]^b^
**FRAP (mg Trolox Equi/g)**	135.600 ± 2.035 [133.297, 137.903]^a^	152.890 ± 1.237 [151.490, 154.290]^b^
**DPPH (mg Trolox Equi/g)**	104.320 ± 1.689 [102.409, 106.231]^a^	121.477 ± 2.276 [118.901, 124.053]^b^
**TAS (mmol/L)**	8.238 ± 0.022 [8.213, 8.263]^a^	8.623 ± 0.013 [8.608, 8.638]^b^
**TOS (µmol/L)**	18.614 ± 0.045 [18.563, 18.665]^a^	19.184 ± 0.057 [19.120, 19.248]^b^
OSI (TOS/(TAS*10))	0.226 ± 0.001 [0.225, 0.227]^b^	0.222 ± 0.001 [0.221, 0.223]^a^

In this study, the antioxidant potentials of optimized extracts obtained from *E. crassipes* species were evaluated comprehensively and the results are presented in Table 2. Both types of extracts obtained with RSM and ANN-GA optimization were analyzed comparatively. The obtained data clearly show that the ANN-GA based extraction protocol provides superior results in terms of antioxidant capacity compared to RSM. The FRAP and DPPH test results, which are functional indicators of antioxidant activity, also showed a similar trend. The FRAP value in ANN-GA extract was measured as 152.890 ± 1.237 mg Trolox/g and the DPPH value as 121.477 ± 2.276 mg Trolox/g; ANN-GA extract was found to be statistically significantly higher than RSM extract in both parameters. These findings confirm that the conditions optimized with the ANN-GA approach are more efficient in terms of iron reducing capacity and free radical scavenging activity. When evaluated in terms of total phenolic content (TPC), it was observed that ANN-GA extract was significantly richer than RSM extract with a value of 209.467 ± 3.590 mg GA equivalent/g (more than 40% increase). Similarly, total flavonoid content (TFC) was measured as 263.867 ± 2.643 mg QE equivalent/g in ANN-GA extract and this value was approximately 33% higher than RSM extract. These results demonstrate that the ANN–GA method can serve as an effective alternative to conventional statistical models for improving extraction performance and bioactivity levels in medicinal plant studies. While previous studies have explored the general bioactivities and chemical compositions of *E. crassipes*^12,13^, they largely relied on conventional extraction methods and lacked comprehensive optimization strategies. For instance, Shanab et al.^12^ focused primarily on allelopathic and antimicrobial effects using crude extracts, without detailing process parameters. Similarly, Ben Bakrim et al.^8^ provided an overview of chemical constituents and traditional uses but did not perform experimental optimization or link extraction efficiency to biological activity. In contrast, the present study applies AI-assisted models (ANN–GA) to systematically optimize extraction conditions and demonstrates significantly higher levels of bioactives and biological effects, thereby providing methodological advancement and novel insights.

These findings are consistent with the information available in the literature on the antioxidant properties of *E. crassipes* extracts. In previous studies, it has been reported that extracts of this species obtained with various solvents (e.g., chloroform, ethanol, aqueous ethanol) exhibited different levels of antioxidant activity by DPPH and FRAP methods^25,26^. In addition, according to the data reported by Shukla et al.^27^, it was observed that total phenolic contents and total flavonoid contents of *E. crassipes* varied between 110 and 372 mg/g and 57.2-452.6 mg/g, respectively, in hexane, chloroform, ethyl acetate, n-butanol and aqueous extracts. In this context, the TPC (209.467 mg/g) and TFC (263.867 mg/g) values ​​obtained in our study are quite high and compatible with the ranges stated in the literature, supporting that the ANN-GA approach is an effective tool in the enrichment of plant bioactives. These similarities may be attributed to the use of comparable solvent systems (ethanol-water mixtures), while observed differences—especially higher TPC and TFC values in our ANN–GA extract—can be explained by the optimization approach employed in the present study, which maximized compound recovery through iterative parameter refinement. Moreover, variations in extraction time, temperature, and plant part used (e.g., whole plant vs. leaf) in previous studies may contribute to the observed discrepancies. The TAS value is also significantly higher in the ANN-GA extract with 8.623 ± 0.013mmol/L compared to the RSM extract (8.238 ± 0.022mmol/L). However, the ANN-GA extract showed a slight increase in TOS values ​​(19.184 ± 0.057µmol/L). However, this situation was not significantly reflected in the oxidative stress index (OSI) value; the OSI values ​​of both extracts were quite close to each other and were calculated as 0.226 and 0.222, respectively. This shows that although the extract obtained with ANN-GA has a higher antioxidant capacity, it maintains a similar redox balance in terms of oxidative load in the system.

No findings were found in the literature regarding the TAS, TOS and OSI values ​​of *E. crassipes*. However, the TAS values ​​of different plant species *Anthemis cotula*, *Mentha longifolia*, *Ulva lactuca* and *Hypericum spectabile* were reported as 7.625, 6.094, 6.135 and 9.306mmol/L, respectively. TOS values ​​were recorded as 11.247, 14.050, 9.761 and 13.065µmol/L, respectively. OSI values ​​​​varyed between 0.148, 0.231, 0.159 and 0.140^20,28–30^. TAS parameter is an important indicator reflecting the general effect of all antioxidant compounds found in natural products^31^. In this context, TAS values ​​​​of both RSM and ANN-GA extracts of *E. crassipes* obtained in our study were found to be higher than *A. cotula*, *M. longifolia* and *U. lactuca* and lower than *H. spectabile*. TOS levels were determined to be higher than all reference plant species. When evaluated in terms of OSI, the values ​​​​of *E. crassipes* extracts; It is higher than *A. cotula*, *U. lactuca* and *H. spectabile*, but lower than *M. longifolia*. OSI is a critical parameter that reveals the percentage suppression of oxidant compounds by antioxidant compounds^31^, and these comparisons provide a quantitative assessment of the antioxidant/oxidant balance potential of *E. crassipes*. In conclusion, the high antioxidant capacity together with the phenolic and flavonoid content of the extract obtained by the ANN-GA based optimization method demonstrate that this approach is an effective and reliable method in the production of functional extracts. These findings, when evaluated together with the results in the literature, indicate that *E. crassipes* is an important source of biologically valuable secondary metabolites and can be evaluated in potential therapeutic applications. In previous studies, antioxidant capacities of *E. crassipes* extracts have been reported in varying degrees depending on solvent type and plant part used. For example, Rabiepour et al.^26^ reported DPPH values ranging from 60.2 to 108.5 mg TE/g depending on ethanol concentration, while FRAP values were generally below 130 mg TE/g. Similarly^27^, found that phenolic content in aqueous-ethanolic extracts of *E. crassipes* ranged between 110 and 170 mg GAE/g. Compared to these findings, the ANN–GA optimized extract in our study exhibited notably higher antioxidant parameters (DPPH: 121.48 mg TE/g; FRAP: 152.89 mg TE/g; TPC: 209.47 mg GAE/g), suggesting that AI-assisted optimization enhances extraction efficiency and phytochemical recovery. Moreover, antioxidant capacity values observed in this study are also superior to many other aquatic plant extracts reported in the literature, including *Nelumbo nucifera*, *Lemna minor*, and *Pistia stratiotes*, which have reported TAS values generally below 8.0 mmol/L^32,33^. These comparative results support the conclusion that the extraction strategy used in this work provides a significant improvement in antioxidant bioefficacy.

### Anticholinesterase activity

Aquatic plants attract attention not only with their ecological roles but also with their pharmacological potential thanks to the bioactive compounds they contain. Phenolic and alkaloid compounds that can inhibit acetylcholinesterase (AChE) and butyrylcholinesterase (BChE) enzymes have been identified in some aquatic plant species^34^. Thanks to these properties, aquatic plants can be evaluated as natural cholinesterase inhibitor sources in the treatment of neurodegenerative diseases^35^. In this study, the cholinesterase enzyme inhibition capacities of optimized extracts obtained from the *E. crassipes* species were evaluated. The IC₅₀ values ​​calculated as a result of the analyzes are presented in detail in Table 3.

**Table 3:** Anticholinesterase activity of *E. crassipes*. Means in the same column with different superscript letters are significantly different (*p* < 0.05) according to duncan’s multiple range test. All cohen`s D were observed very high (> 1.4).

Sample	AChEµg/mL Mean ± [95%CI]	BChEµg/mL Mean ± [95%CI]
**Galantamine**	7.710 ± 0.281 [7.392, 8.028]^a^	16.830 ± 0.196 [16.608, 17.052]^a^
**RSM extract**	72.417 ± 1.626 [70.577, 74.257]^c^	88.303 ± 1.190 [86.956, 89.650]^c^
**ANN-GA extract**	61.687 ± 1.696 [59.768, 63.606]^b^	81.397 ± 1.238 [79.996, 82.798]^b^

In our study, anticholinesterase effects of optimized extracts of *E. crassipes* species were also evaluated. IC₅₀ values ​​determined for the reference inhibitor galantamine against AChE and BChE enzymes were determined as 7.710 ± 0.281 µg/mL and 16.830 ± 0.196 µg/mL, respectively. While the IC₅₀ values ​​of the extract optimized with RSM were 72.417 ± 1.626 µg/mL for AChE and 88.303 ± 1.190 µg/mL for BChE; the extract obtained with ANN-GA optimization showed higher inhibitory effect with lower IC₅₀ values ​​(AChE: 61.687 ± 1.696 µg/mL; BChE: 81.397 ± 1.238 µg/mL). These results show that the extract obtained with the ANN-GA protocol is more effective not only in terms of antioxidant capacity but also in terms of its inhibitory potential on cholinesterases, which are neurological target enzymes. The findings obtained support previous studies on the anticholinesterase effects of *E. crassipes*. Indeed, Adeleke et al.^36^ reported that this plant can inhibit the acetylcholinesterase enzyme. In this direction, the IC₅₀ values ​​obtained in our study coincide with the findings in the literature and quantitatively confirm the cholinesterase inhibitory potential of *E. crassipes*. Natural compounds with anticholinesterase activity are among the priority research targets in the treatment of neurodegenerative diseases, especially those with cholinergic neurotransmission disorders such as Alzheimer’s disease^37^. The inhibitory effect of extracts obtained from *E. crassipes* on cholinesterase enzymes indicates that this species contains not only antioxidant capacity but also secondary metabolites that may have potential therapeutic effects on the central nervous system^38^. This makes it possible to evaluate this low-cost and widespread aquatic plant among sustainable and natural cholinesterase inhibitor sources.

### Antiproliferative activity

**Fig. 3:**
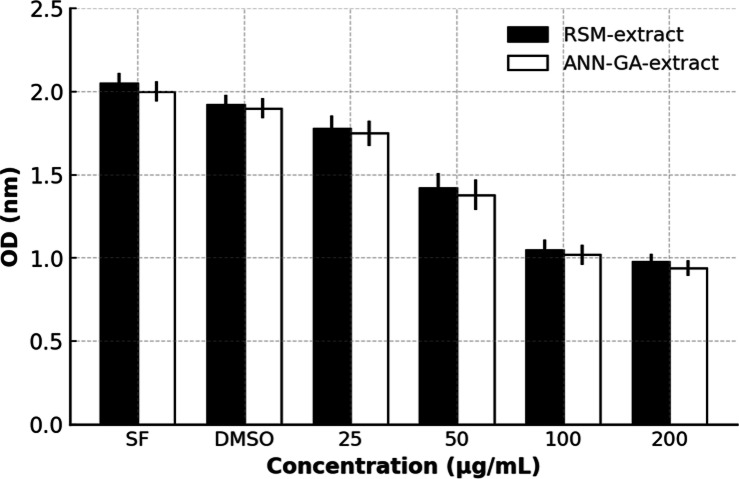
Antiproliferative activity of *E. crassipes*.

Some aquatic plants can exhibit antiproliferative effects thanks to secondary metabolites such as phenolics, flavonoids, and alkaloids found in their natural compounds. Extracts obtained from these plants can suppress tumor cell growth by inhibiting cell division in various cancer cell lines. This feature of aquatic plants makes them important and promising candidates in studies aimed at the development of natural anticancer agents^39,40^. In this direction, in our study, the antiproliferative activity of the optimized extract obtained from *E. crassipes* on the A549 human lung cancer cell line was evaluated. The findings are visually presented in Fig. 3.

In our study, the antiproliferative effects of optimized extracts obtained from *E. crassipes* on human lung adenocarcinoma cell line (A549) were evaluated. While the control group consisted of cells incubated only in culture medium, the DMSO group was treated only with medium containing DMSO in order to evaluate the effect of the extraction solvent. In the application groups, extracts obtained with the RSM and ANN-GA protocol were applied to the cells at concentrations of 25, 50, 100 and 200 µg/mL, respectively. It was observed that both extracts significantly reduced cell viability depending on the concentration. The fact that the DMSO group had optical density (OD) values ​​close to the control group indicates that the solvent did not have a significant toxic effect on cell proliferation. On the other hand, the ANN-GA extract applied especially at concentrations of 100 and 200 µg/mL produced lower OD values ​​compared to the RSM extract, and this was associated with a stronger antiproliferative effect. These findings suggest that the extract obtained with ANN-GA optimization may contain more biologically effective components and have a stronger suppressive effect on cancer cells. There are similar findings in the literature; *E. crassipes* has previously been reported to have an antiproliferative effect on HeLa cells by Lenora et al.^41^. In addition, in a study conducted by Kumar et al.^42^, it was reported that the water extract of this plant exhibited antiproliferative activity on breast (T47D), prostate (PC3) and lung (NCI-H322 and A549) cancer cell lines. These literature supports are compatible with the findings obtained in our study and support that *E. crassipes* may be a potential natural antitumor agent against a wide variety of cancer types. In this context, the importance of extract optimization becomes clear. The biological effects of plant-derived bioactive compounds are closely related not only to species diversity but also to extraction parameters^43^. Advanced optimization approaches such as ANN-GA can increase pharmacological activity by improving the phytochemical profile and bioequivalence of the extract. This study shows that determining the appropriate extraction strategy directly affects the therapeutic potential of the extract to be obtained; it proves that optimized extracts can create stronger biological responses compared to crude or randomly obtained extracts. In conclusion, *E. crassipes* can be considered as a valuable natural resource in terms of pharmacology not only with its antioxidant and anticholinesterase potential but also with the high antiproliferative effect exhibited by its optimized extracts. Although IC₅₀ values were not calculated due to the limited concentration range, cell viability percentages were estimated based on normalized OD values to facilitate comparison with previous studies. In addition, although the present study did not experimentally assess apoptotic or cell cycle arrest pathways, previous reports have shown that phenolic compounds abundantly identified in the ANN–GA extract—such as quercetin, kaempferol, and naringenin—can induce apoptosis and inhibit cell proliferation via multiple signaling cascades, including mitochondrial-mediated pathways and cell cycle regulation. These known effects suggest that the antiproliferative activity observed in our extract may be partially attributed to such mechanisms.

However, the absence of IC₅₀ values—resulting from the limited concentration range tested—restricts direct numerical comparison with previous studies. Likewise, while literature data suggest potential mechanisms such as apoptosis induction and cell cycle arrest mediated by phenolic constituents (e.g., quercetin, kaempferol, naringenin), these pathways were not experimentally confirmed in the present work. Future research should therefore incorporate broader concentration ranges for IC₅₀ determination and mechanistic assays (e.g., apoptosis or cell cycle analyses) to strengthen the pharmacological interpretation of antiproliferative effects.

### Phenolic contents

Aquatic plants are among the natural resources rich in phenolic compounds, and these compounds play an important role in the defense of the plant against oxidative stress, ultraviolet rays and microbial attacks. Therefore, aquatic plants stand out as potential phytochemical reservoirs that can be evaluated in the pharmaceutical, food and cosmetic industries^32,44^. In this context, in our study, the phenolic compound profile of the optimized extracts of *E. crassipes* was analyzed using LC-MS/MS device. The qualitative and quantitative findings are presented in detail in Table 4.

**Table 4:** Phenolic contents of *E. crassipes*. Means in the same row with different superscript letters are significantly different (*p* < 0.05) according to the independent t-test. All cohen`s D were observed high (> 1.4).

Phenolic compound	RSM extract (mg/kg) Mean ± [95%CI]	ANN-GA extract (mg/kg) Mean ± [95%CI]
**Kaempferol**	8373.35 ± 6.20 [8366.33, 8380.37]^a^	8656.31 ± 2.41 [8653.58, 8659.04] ^b^
**Gallic acid**	3131.69 ± 15.33 [3114.34, 3149.04] ^a^	4969.18 ± 2.30 [4966.58, 4971.78] ^b^
**4-hydroxybenzoic acid**	358.52 ± 4.70 [353.20, 363.84] ^a^	1680.16 ± 1.55 [1678.41, 1681.91] ^b^
**Naringenin**	4164.32 ± 5.73 [4157.84, 4170.80] ^a^	5364.56 ± 3.77 [5360.29, 5368.83] ^b^
**Quercetin**	7162.80 ± 2.78 [7159.65, 7165.95] ^a^	10295.26 ± 2.77 [10292.13, 10298.39] ^b^
**Luteolin**	2165.30 ± 7.38 [2156.92, 2173.68] ^b^	2091.35 ± 2.29 [2088.84, 2093.86] ^a^
**Catechinhyrate**	966.24 ± 2.34 [963.59, 968.89] ^b^	527.82 ± 1.57 [525.99, 529.65] ^a^
**Myricetin**	2649.96 ± 2.48 [2647.15, 2652.77] ^b^	2935.96 ± 2.68 [2933.05, 2938.87] ^a^

**Fig. 4:**
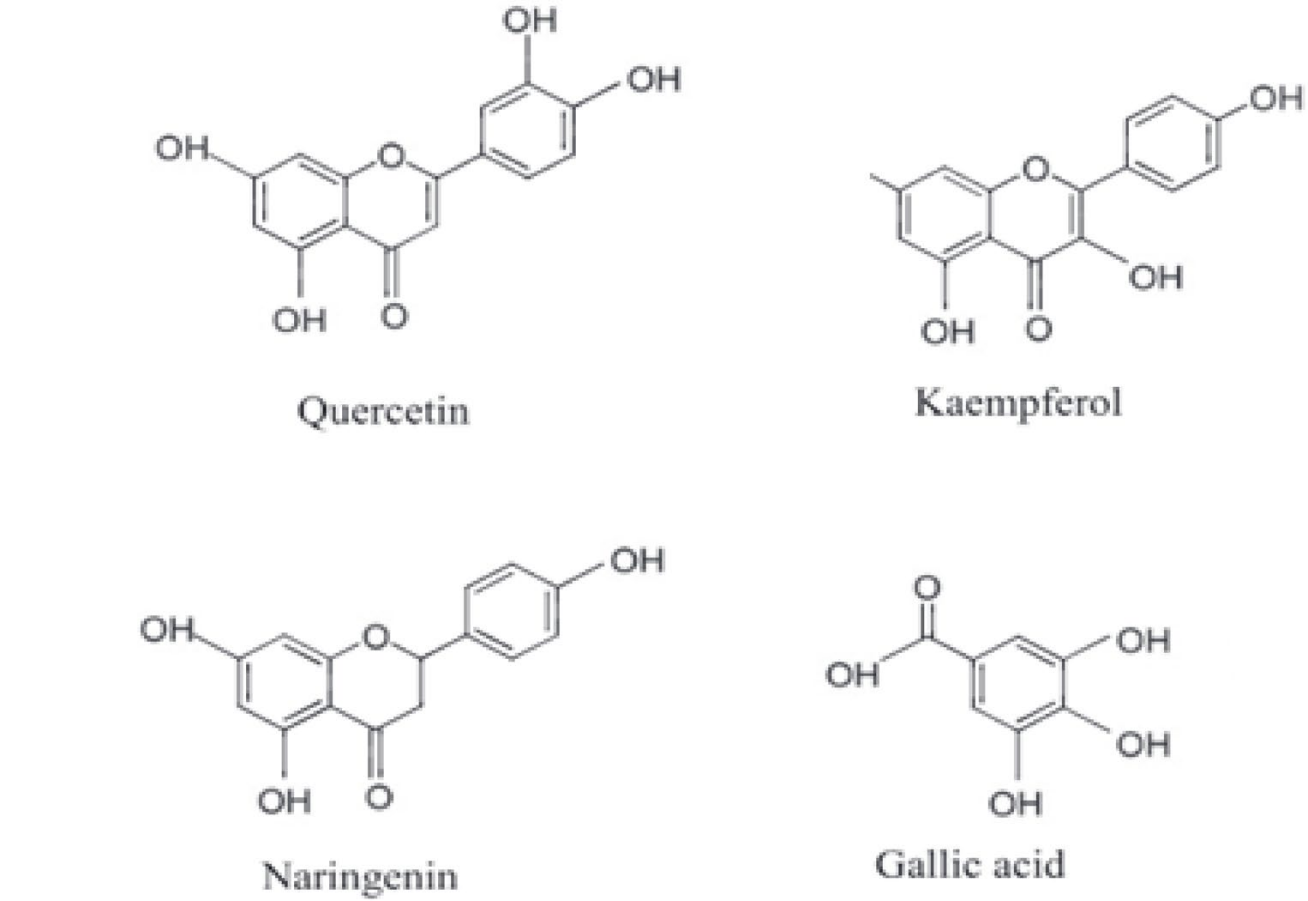
Chemical structures of major phenolic compounds identified in the optimized *E. crassipes* extract, including quercetin, kaempferol, naringenin and gallic acid^45^.

The chemical structures of the most abundant phenolic compounds identified in the optimized extract are shown in Fig. 4.

In this study, the phenolic compound contents of extracts obtained from *E. crassipes* using different optimization strategies (RSM and ANN–GA) were analyzed by LC-MS/MS. Among these, quercetin was identified as the most abundant phenolic compound, reaching 10,295.26 mg/kg in the ANN–GA optimized extract, a significantly higher value compared to the RSM extract. Quercetin is known to inhibit apoptosis and inflammation pathways by reducing intracellular oxidative stress^5^, suggesting that the ANN–GA model facilitates the efficient release of large and complex flavonols. Similarly, kaempferol and naringenin were also found at elevated levels in the ANN–GA extract (8656.31 mg/kg and 5364.56 mg/kg, respectively), highlighting the model’s effectiveness in enhancing total flavonoid recovery. Naringenin is associated with antioxidant and hepatoprotective properties^6^, while kaempferol is known to suppress cancer cell proliferation and promote cardiovascular health^7^.

Phenolic acids such as gallic acid and 4-hydroxybenzoic acid were also significantly increased in the ANN–GA extract. Notably, the concentration of 4-hydroxybenzoic acid was approximately fivefold higher than that in the RSM extract (1680.16 mg/kg), indicating enhanced solubility of polar compounds through ANN–GA-assisted cell wall disruption. Gallic acid, with its strong antioxidant, antimicrobial, and antimutagenic properties^9^, was also substantially enriched. On the other hand, catechinhydrate was found at lower levels in the ANN–GA extract, underscoring that extraction efficiency may vary depending on the structural and polarity characteristics of individual phenolics. Myricetin levels were comparable across both extraction strategies, though slightly higher in the ANN–GA method. Known for its neuroprotective and anti-inflammatory effects^8^, myricetin further supports the potential health relevance of the extract.

Overall, the ANN–GA model was demonstrated to be effective in maximizing the production of bioavailable phenolic compounds. Although formal statistical correlation analysis was not performed, comparative evaluation of the phenolic profiles and bioactivity data suggests that quercetin, kaempferol, gallic acid, and naringenin are likely major contributors to the observed antioxidant and anticholinesterase effects. Their high concentrations in the ANN–GA optimized extract are consistent with the enhanced FRAP and DPPH values and lower IC₅₀ values for enzyme inhibition, supporting their probable role in the extract’s pharmacological efficacy. These findings underline the utility of ANN–GA as an advanced and robust approach in plant-based extraction technologies. However, due to the limited number of quantified minor constituents, a meaningful correlation analysis between individual compounds and biological activities was not feasible in this study. This represents a limitation, and future research should aim to include a broader range of phytochemicals to enable robust statistical correlation.

## Conclusion

This study provides a comprehensive evaluation of the biological activities of *Eichhornia crassipes* (water hyacinth) extracts obtained under optimized extraction conditions. The Artificial Neural Network–Genetic Algorithm (ANN–GA) optimization method yielded extracts with higher phenolic and flavonoid contents, stronger antioxidant capacity, and a more pronounced antiproliferative effect compared to the conventional Response Surface Methodology (RSM). In addition, inhibitory effects on cholinesterase enzymes were more prominent in ANN–GA extracts. LC–MS/MS analyses revealed that extracts obtained with ANN–GA possessed a richer and pharmacologically valuable phenolic compound profile. These findings highlight the potential of *E. crassipes* as a natural, sustainable, and economically accessible bioactive source for applications in the pharmaceutical, food, and cosmetic industries.

The economic accessibility of E. crassipes derives from its exceptional abundance and rapid growth in nutrient-rich freshwater systems, where it is often harvested as part of ecological management programs. In contrast to cultivated medicinal plants, it can be collected directly from natural water bodies without requiring agricultural land, irrigation, or intensive cultivation inputs. These characteristics substantially reduce biomass acquisition costs compared with terrestrial plant sources, supporting its feasibility as a cost-effective raw material for large-scale bioactive compound production.

Specifically, the optimized extract obtained via ANN–GA demonstrated significantly higher biological efficacy, with TPC and TFC values of 209.47 mg GAE/g and 263.87 mg QE/g, respectively. Antioxidant capacity was notably improved, with FRAP and DPPH activities of 152.89 mg TE/g and 121.48 mg TE/g, respectively. Moreover, the extract exhibited strong anticholinesterase activity, with IC₅₀ values of 61.69 µg/mL (AChE) and 81.40 µg/mL (BChE), and a marked dose-dependent antiproliferative effect against A549 cells. Overall, these results confirm that AI-assisted optimization provides a robust and innovative strategy for enhancing the bioactivity and pharmacological potential of plant-based extracts.

Beyond the comparative findings between ANN–GA and RSM, these results may have broader implications for industrial phytochemical extraction. By enabling precise tuning of extraction parameters, the ANN–GA model offers significant potential for scalable, cost-efficient bioprocess development. Its ability to maximize bioactive compound recovery with high reproducibility makes it a valuable tool for pharmaceutical, nutraceutical, and environmental applications. Furthermore, integrating such AI-assisted methods into green chemistry workflows could support the sustainable production of functional plant-based products from aquatic biomass.

Future Perspective: In future studies, the optimized extract should be evaluated in vivo to determine its pharmacokinetic profile and toxicity. Additionally, the development of pharmaceutical or nutraceutical formulations (e.g., capsules, functional foods, or topical applications) could be explored. Applying ANN–GA strategies to other medicinal or aquatic plants may also open new avenues for precision phytochemical extraction and the development of high-value bioproducts.

## Data Availability

The datasets used and/or analyzed during the current study are available from the corresponding author upon reasonable request.
